# Use of the Coding Causes of Death in HIV in the classification of deaths in Northeastern Brazil

**DOI:** 10.11606/S1518-8787.2017051000124

**Published:** 2017-09-11

**Authors:** Diana Neves Alves, Cristiane Campello Bresani-Salvi, Joanna d’Arc Lyra Batista, Ricardo Arraes de Alencar Ximenes, Demócrito de Barros Miranda, Heloísa Ramos Lacerda de Melo, Maria de Fátima Pessoa Militão de Albuquerque

**Affiliations:** I Programa de Pós-Graduação em Saúde Pública. Centro de Pesquisas Aggeu Magalhães. Fundação Oswaldo Cruz. Recife, PE, Brasil; II Universidade Federal da Fronteira Sul. Chapecó, SC, Brasil; III Departamento de Medicina Tropical. Universidade Federal de Pernambuco. Recife, PE, Brasil; IV Faculdade de Ciências Médicas. Universidade de Pernambuco. Recife, PE, Brasil; V Centro de Ciências da Saúde. Universidade Federal de Pernambuco. Recife, PE, Brasil; VI Departamento de Saúde Coletiva. Centro de Pesquisas Aggeu Magalhães. Fundação Oswaldo Cruz. Recife, PE, Brasil

**Keywords:** Acquired Immunodeficiency Syndrome, mortality, Cause of Death, AIDS-Related Opportunistic Infections, mortality

## Abstract

**OBJECTIVE:**

Describe the coding process of death causes for people living with HIV/AIDS, and classify deaths as related or unrelated to immunodeficiency by applying the Coding Causes of Death in HIV (CoDe) system.

**METHODS:**

A cross-sectional study that codifies and classifies the causes of deaths occurring in a cohort of 2,372 people living with HIV/AIDS, monitored between 2007 and 2012, in two specialized HIV care services in Pernambuco. The causes of death already codified according to the International Classification of Diseases were recoded and classified as deaths related and unrelated to immunodeficiency by the CoDe system. We calculated the frequencies of the CoDe codes for the causes of death in each classification category.

**RESULTS:**

There were 315 (13%) deaths during the study period; 93 (30%) were caused by an AIDS-defining illness on the Centers for Disease Control and Prevention list. A total of 232 deaths (74%) were related to immunodeficiency after application of the CoDe. Infections were the most common cause, both related (76%) and unrelated (47%) to immunodeficiency, followed by malignancies (5%) in the first group and external causes (16%), malignancies (12 %) and cardiovascular diseases (11%) in the second group. Tuberculosis comprised 70% of the immunodeficiency-defining infections.

**CONCLUSIONS:**

Opportunistic infections and aging diseases were the most frequent causes of death, adding multiple disease burdens on health services. The CoDe system increases the probability of classifying deaths more accurately in people living with HIV/AIDS.

## RESUMO

**OBJETIVO:**

Descrever o processo de codificação das causas de morte em pessoas vivendo com HIV/Aids, e classificar os óbitos como relacionados ou não relacionados à imunodeficiência aplicando o sistema *Coding Causes of Death in HIV* (*CoDe*).

**MÉTODOS:**

Estudo transversal, que codifica e classifica as causas dos óbitos ocorridos em uma coorte de 2.372 pessoas vivendo com HIV/Aids acompanhadas entre 2007 e 2012 em dois serviços de atendimento especializado em HIV em Pernambuco. As causas de óbito já codificadas a partir da Classificação Internacional de Doenças foram recodificadas e classificadas como óbitos relacionados e não relacionados à imunodeficiência pelo sistema *CoDe*. Foram calculadas as frequências dos códigos *CoDe* das causas do óbito em cada categoria de classificação.

**RESULTADOS:**

Ocorreram 315 (13%) óbitos no período do estudo; 93 (30%) tinham como causa uma doença definidora de Aids da lista do *Centers for Disease Control and Prevention*. No total 232 óbitos (74%) foram relacionados à imunodeficiência após aplicar o *CoDe*. As infecções foram as causas mais comuns, tanto nos óbitos relacionados (76%) como não relacionados (47%) à imunodeficiência, seguindo-se de malignidades (5%) no primeiro grupo e de causas externas (16%), malignidades (12%) e doenças cardiovasculares (11%) no segundo. A tuberculose compreendeu 70% das infecções definidoras de imunodeficiência.

**CONCLUSÕES:**

Infecções oportunistas e doenças do envelhecimento foram as causas mais frequentes de óbito, imprimindo carga múltipla de doenças aos serviços de saúde. O sistema *CoDe* aumenta a probabilidade de classificar os óbitos com maior precisão em pessoas vivendo com HIV/Aids.

## INTRODUCTION

The AIDS pandemic between the 1970s and 1980s was marked by the emergence of previously rare opportunistic infectious-parasitic diseases, as well as the emergence of new diseases specific to HIV infection and its treatment^6^. Since the 1990s, after the advent of combined antiretroviral therapy (ART), there has been a progressive reduction in mortality, diversification of causes of death, and increased frequency of non-immunodeficient deaths^18^ ^,^ ^19^. In Brazil, these changes began in 1996^1^ ^,^ ^18^, when ART was introduced in the Brazilian Unified Health System (SUS). However, mortality rates among people living with HIV/AIDS (PLWHA) are still higher than the general population rates, and immunodeficiency-related diseases, led by tuberculosis, continue to contribute significantly to deaths in developed^9^ ^,^ ^10^ and developing^8^ ^,^ ^13^ countries.

The monitoring of causes of death related or not to immunodeficiency is important for targeted interventions to increase the survival of PLWHA. However, the heterogeneity of coding and classification methods makes it difficult to compile and compare studies^15^. The World Health Organization’s International Classification of Diseases (ICD-10) system is widely used but does not cover some AIDS-defining diseases and adverse effects of ART^1^ ^,^ ^10^. With this concern, a consensus of experts launched, in 2004, the Coding Causes of Death in HIV Protocol (CoDe)^4^, a standardized international coding and classification system for deaths. The CoDe comprises two processes. The first uses the Case Report Form to collect sociodemographic and clinical data of the case, and the second consists of a matched review to determine the sequence of events that led to death and classifies the death as related or not to immunodeficiency through the Review Form^4^.

Hernando et al.^9^ applied algorithms based on CoDe and ICD-10. These authors related 53% of deaths to immunodeficiency in their sample when using the CoDe system; while, when using ICD-10, the percentage would be 70%, since ill-defined causes, hepatitis and infections, particularly pneumonia, are attributed to HIV/AIDS in PLWHA deaths. Although these differences may generate biases and classification errors, the CoDe’s tools have not been systematically applied in research practice. Two studies used the CoDe methodology to classify deaths in PLWHA in Southeast Brazil, both reporting a time trend of reduced mortality from AIDS-related causes^1^ ^,^ ^8^.

We did not find publications on the classification and profile of PLWHA deaths in northeastern Brazil. However, we believe that AIDS infections are significant causes of deaths in our region, where there are low ART coverage and endemic rates of tuberculosis. Therefore, this study aimed to describe the causes of death among people living with HIV/AIDS in a northeastern capital and to classify them as to their relationship with HIV immunodeficiency by means of the Coding Causes of Death in HIV Protocol method.

## METHODS

This is a cross-sectional study that describes and classifies the causes of deaths occurring in a cohort of PLWHA monitored by two specialized HIV care services (SAE) of SUS in the city of Recife, State of Pernambuco, responsible for the care of about 70% of PLWHA in the state at the time of recruitment. The study population consisted of a cohort of 2,372 patients recruited between July 2007 and June 2010. Participants were eligible if they were seropositive for HIV reagent, SAE patients, and older than 17 years. We did not establish exclusion criteria.

Information on the basic, intermediate and contributing cause of death was obtained from the information contained in the death certificates registered in the Mortality Information System of Pernambuco (SIM-PE). Monitoring and identification of cohort deaths in the SIM were done until December 2012, using probabilistic linkage through the RecLink III2 program^2^. A database was created with cohort deaths and each case was coded and classified as related or unrelated to immunodeficiency, according to CoDe Protocol^4^.

The scientific use of CoDe instruments exempts permission or payment of fees. The production of publications with the CoDe methodology is encouraged by the coordination of the CoDe Project, which only requests that it be informed of its use in the methods session, referencing the website^a^. As part of the present study, the CoDe instruments were translated by specialists and their respective instructions for completion in the Brazilian Portuguese language. The instruments were made available for free use in supplementary materials: Case Report Form (HIV Death Report Form – ReCoDe) and Review Form (HIV Death Revision and Coding Form – RevCoDe).

This study was based on a secondary database containing the causes of death recorded with ICD-10 codes, as captured in SIM-PE. Therefore, we did not apply the Case Report Form stage, but we translated it (ReCoDe) and tested it on two new cases of death in the Cancer Hospital in Pernambuco, based on their medical records and death certificate. Considering the problem of underreporting in health information systems and endemic tuberculosis in our region, we adapted the ReCoDe with the inclusion of fields for data collection on the causes of death in the death certificate and SIM (section 5, Table 1) and about treatment for tuberculosis (section 7, Table 2). These adaptations did not detract from the content of the original instruments and were duly informed in addendums in the translated versions (see additional materials)^b^ *.*

**Table 1:** Frequencies of the causes of death according to the Coding Causes of Death in HIV Protocol system among 232 deaths related to the immunodeficiency of people living with HIV/AIDS. Recife, State of Pernambuco, 2007–2012.

Causes of death CoDe	ICD-10 basic causes of death	n	%
01 AIDS (active disease in progress)	B22.0; B22.2; B22.7	8	3.4
01.1 Infection-AIDS	A41.9; B20; B20.0; B20.1; B20.2; B20.5; B20.6; B20.7; B20.8; B22.7	73	31.5
01.2 Cancer-AIDS	B20.8; B21.0; B21.2; B22.7; C53.9; C85.9	12	5.2
02 Infection (except 01.1)	-	-	-
02.1 Bacterial	B20.1	1	0.4
02.1.1 Bacterial with sepsis	A41.9; B20.1; B20.7; B22.7; J18.2	43	18.5
02.2 Others	B24	1	0.4
02.2.1 Other with sepsis	-	-	-
02.3 Etiology unknown	A09; B20.7; B20.8; B20.9; B22.7	36	15.5
02.3.1 Unknown with sepsis	B20.7; B22.7	22	9.5
03 Chronic viral hepatitis (progression/complication)	-	-	-
03.1 HCV	-	-	-
03.1.1 HCV with cirrhosis	-	-	-
03.1.2 HCV with liver failure	B22.7	1	0.4
03.2 HBV	-	-	-
03.2.1 HBV with cirrhosis	-	-	-
03.2.2 HBV with liver failure	-	-	-
04 Cancer (except 01.2, 03, 03.1, 03.2)	B21.7; B21.8; B22.7; C21.1; C34.9; C80	7	3.0
05 Diabetes Mellitus (complication)	-	-	-
06 Pancreatitis	-	-	-
07 Lactic acidosis	-	-	-
08 MI or other ischemic heart disease	-	-	-
08.1 Definitive MI (Dundee 1)	-	-	-
08.2 Possible MI (Dundee 2/9)	-	-	-
08.3 Other ischemic heart disease	-	-	-
09 Stroke	-	-	-
10 Gastrointestinal bleeding	K92.0	1	0.4
11 Primary pulmonary hypertension	-	-	-
12 Pulmonary embolism	-	-	-
13 Chronic obstructive pulmonary disease	-	-	-
14 Hepatic insufficiency (except 03, 03.1, 03.2)	-	-	-
15 Renal insufficiency	B24	2	0.9
16 Accident or other violent death (not suicide)	-	-	-
17 Suicide	-	-	-
18 Euthanasia	-	-	-
19. Substance abuse (active)			
19.1 Chronic alcohol abuse	K70.3	1	0.4
19.2 Chronic intravenous drug use	-	-	-
19.3 Acute intoxication	-	-	-

General classification when the cause of death could not be specifically classified			

20 Other hematological causes	B22.7	1	0.4
21 Other endocrine causes	-	-	-
22 Other psychiatric causes	-	-	-
23 Other CNS causes	-	-	-
24 Other cardiovascular causes	I50.0; I51.4; I51.7; I67.8	4	1.7
25 Other respiratory causes	-	-	-
26 Other digestive system causes	K83.0	1	0.4
27 Other dermatological or motor causes	-	-	-
28 Other urogenital causes	N12	1	0.4
29 Obstetric complications	-	-	-
30 Congenital diseases	-	-	-

Unclassifiable causes of death			

90 Other causes	-	-	-
91 Unclassifiable causes	B22.7; B23.8; B24	15	6.5
92 Unknown causes	R98; Y34.9	2	0.9

Total	-	232	100

CoDe: Coding Causes of Death in HIV Protocol; PLWHA: People living with HIV/AIDS; ICD-10: Tenth International Classification of Diseases; HCV: Hepatitis C Virus; HBV: Hepatitis B Virus; MI: Myocardial infarction; CNS: Central Nervous System

**Table 2:** Frequencies of the causes of death according to the Coding Causes of Death in HIV Protocol system among 83 deaths related to the immunodeficiency of people living with HIV/AIDS. Recife, State of Pernambuco, 2007–2012.

Cause of death CoDe	ICD-10 basic cause of death	n	%
01 AIDS (active disease in progress)	-	-	-
01.1 Infection-AIDS	-	-	-
01.2 Cancer-AIDS	-	-	-
02 Infection (except 01.1)	-	-	-
02.1 Bacterial	M60.0	1	1.2
02.1.1 Bacterial with sepsis	B20.1; B20.7; G00.9; K65.0; K81.9	20	24.1
02.2 Others	A91; B20.3	2	2.4
02.2.1 Other with sepsis	-	-	-
02.3 Etiology unknown	B20.7; B20.8	12	14.5
02.3.1 Unknown with sepsis	B20.7; J98.8	4	4.8
03 Chronic viral hepatitis (progression/complication)	-	-	-
03.1 HCV	-	-	-
03.1.1 HCV with cirrhosis	-	-	-
03.1.2 HCV with liver failure	-	-	-
03.2 HBV	-	-	-
03.2.1 HBV with cirrhosis	-	-	-
03.2.2 HBV with liver failure	-	-	-
04 Cancer (except 01.2, 03, 03.1, 03.2)	B21.3; B21.8; C02.9; C21.1; C23; C34.9; C49.9; C50.9; C56	10	12.0
05 Diabetes Mellitus (complication)	E14.9	1	1.2
06 Pancreatitis	-	-	-
07 Lactic acidosis	-	-	-
08 MI or other ischemic heart disease	I21.9	4	4.8
08.1 Definitive MI (Dundee 1)	-	-	-
08.2 Possible MI (Dundee 2/9)	-	-	-
08.3 Other ischemic heart disease	-	-	-
09 Stroke	-	-	-
10 Gastrointestinal bleeding	-	-	-
11 Primary pulmonary hypertension	-	-	-
12 Pulmonary embolism	-	-	-
13 Chronic obstructive pulmonary disease	-	-	-
14 Hepatic insufficiency (except 03, 03.1, 03.2)	K72.9; K76.9	2	2.4
15 Renal insufficiency	I12.0	1	1.2
16 Accident or other violent death (not suicide)	V09.3; X95.4; X95.9; X99.9; Y00.4; Y20.0; Y34.9	13	15.7
17 Suicide	-	-	-
18 Euthanasia	-	-	-
19. Substance abuse (active)	K70.1	1	1.2
19.1 Chronic alcohol abuse	-	-	-
19.2 Chronic intravenous drug use	-	-	-
19.3 Acute intoxication	-	-	-

General classification when the cause of death could not be specifically classified			

20 Other hematological causes	-	-	-
21 Other endocrine causes	-	-	-
22 Other psychiatric causes	-	-	-
23 Other CNS causes	B24	1	1.2
24 Other cardiovascular causes	B24; I11.9; I42.0; I50.0	5	6.0
25 Other respiratory causes	-	-	-
26 Other digestive system causes	K29.1	1	1.2
27 Other dermatological or motor causes	-	-	-
28 Other urogenital causes	-	-	-
29 Obstetric complications	-	-	-
30 Congenital diseases	-	-	-

Unclassifiable causes of death			

90 Other causes	-	-	-
91 Unclassifiable causes	B22.7; B24	3	3.6
92 Unknown cause	R98; Y34.0	2	2.4

Total	-	83	100

CoDe: Coding Causes of Death in HIV Protocol; PLWHA: People living with HIV/AIDS; ICD-10: Tenth International Classification of Diseases; HCV: Hepatitis C Virus; HBV: Hepatitis B Virus; MI: Myocardial infarction; CNS: Central Nervous System

Therefore, we performed only the RevCo step according to the clinical judgment of the immediate, intermediate, contributing, and basic causes in the study database and a code was then assigned to each case among the 30 categories of the CoDe system. Death was coded with CoDe 01 (AIDS-active disease at the time) if an intermediate, contributing, or basic cause was an infection (CoDe 01.1) or cancer (CoDe 01.2) on the CoDe’s list of AIDS-defining diseases. Other bacterial infections that did not meet CoDe 01.1 definitions were coded as other bacterial infections (CoDe 02.1) or other bacterial infections with sepsis (CoDe 02.1.1) if there were any ICD-10 cause referring to sepsis. Deaths described as due to sepsis or septicemia, but without mention of AIDS-defining conditions or bacterial infections were not included in CoDe 01.1 or 02.1.1, and were coded as other infections with sepsis (CoDe 02.2.1).

Deaths attributed to liver failure, cirrhosis or liver neoplasia in the underlying cause were classified as chronic viral hepatitis (CoDe 03) if chronic hepatitis B or C had been mentioned in the intermediate causes. Otherwise, the deaths were coded as liver failure (CoDe 14). Deaths described as due to neoplasia and that did not fit CoDe 01.2 (AIDS cancer) or the CoDe 03 group (chronic viral hepatitis) were classified as other cancers (CoDe 04).

Death was coded as CoDe 08 (ischemic heart disease) if the underlying, intermediate, or contributing cause belonged to the ICD-10 ischemic heart disease group (I20 to I25), or if the underlying cause was nonspecific heart disease, but one of the intermediary causes belonged to this ICD-10 group.

Deaths with ICD-10 of external causes in the basic cause were classified as an accident or other violent deaths (CoDe 16), however, when there was mention of suicide, the attributed CoDe was 17. When, according to ICD-10, the cause was an unspecified HIV disease, without mention of other diagnoses, we attribute it CoDe 91 (unclassifiable causes). Deaths described as due to cardiac arrest or respiratory failure without other information, or those with insufficient information to reach a consensus were coded as due to unknown cause (CoDe 92).

Deaths from external causes were considered to be unrelated to immunodeficiency, while cases that had an AIDS-defining condition on the CoDe list (the modified list of the Centers for Disease Control and Prevention)^3^ or Hodgkin’s lymphoma as the underlying, intermediate, or contributing cause were classified as definitely related to immunodeficiency. The other cases were all considered as non-sudden deaths and thus classified as related or not to immunodeficiency depending on the CD4 lymphocyte count < or ≥ 200 cells/mm^3^, respectively. For this purpose, we evaluated the CD4 lymphocyte count closer to death in the year before death. The 10 cases that did not have information on CD4 count and the 47 cases whose last CD4 result dated more than 365 days before death were classified only by the clinical judgment of the causes of death.

Two independent reviewers (CCB and JDLB) filled the fields of intermediate cause, the four contributing causes and the basic cause in the RevCoDe form, respectively, with the immediate cause, the two intermediate causes and two contributing causes, and with the underlying basic cause as YES; and they later proceeded to the process of classification of death as related and unrelated to immunodeficiency case by case. Disagreements between the two reviewers (nine cases) were sent to a third party (MFPMA) for the final classification. We used the Microsoft® Excel 2010 program to calculate the frequencies of the CoDe codes for the causes of death in each classification category: related and unrelated to immunodeficiency.

All participants read and signed the informed consent form for inclusion in the cohort. This study was approved by the Ethics Committee of the Universidade Federal de Pernambuco (CEP/CCS/UFPE 254/05).

## RESULTS

The studied population included 315 (13.3%) deaths occurring in a cohort of 2,372 PLWHA. We classified a total of 232 deaths (73.6%) as immunodeficiency-related, 93 (29.5%) of which were based on the identification of an AIDS-defining disease among the basic, intermediate, or contributory causes recorded in the SIM, and 139 according to the CoDe algorithm. In the group of deaths due to immunodeficiency, 40% of the patients were aged ≥ 40 years, while among the non-immunodeficient deaths, this percentage was 70%. Male patients accounted for 70% of the population. In both groups, approximately half of the patients had a monthly income below the minimum wage, the non-literate were 80%, the unemployed 80% and smokers or former smokers 60%. Regarding drug use, 30% of patients consumed alcohol and 30% had a history of illicit drug use.

In the group of deaths related to immunodeficiency group (Table 1), AIDS cancers represented 5.2% of the events; unclassifiable or unknown causes (CoDe 91 and 92) accounted for 7.4%, and AIDS (31.5%) and non-AIDS infections (44.4%) were the most frequent causes. ICD-10 codes related to tuberculosis contributed to 68.5% of deaths from AIDS infection. Infectious diseases were also the most frequent among the 83 non-immunodeficient deaths (47%), followed by an accident or another violent death (15.7%), except for suicide. Non-AIDS cancers accounted for 12%, cardiovascular diseases were 10.8%, and unclassifiable or unknown causes were 6% of deaths not related to immunodeficiency, according to Table 2.

The ICD-10 codes that refer to HIV infection (B20 to B24) were recorded in the basic cause of 251 deaths (80%), 30 of which were not related to immunodeficiency after applying the CoDe algorithm. In 41 cases (13%) there was no record of HIV infection in any cause of death of the SIM (immediate, intermediate, contributing, and basic). However, 11 of these cases were related to immunodeficiency after the CoDe algorithm was applied.

## DISCUSSION

We were able to classify all 315 deaths as related or not to PLWHA immunodeficiency, using the CoDe^4^ algorithm presented here and translated into Brazilian Portuguese for the first time. Twenty-two deaths received a CoDe code of unknown or unclassifiable cause, reflecting insufficient information about the causa mortis. Less than half of the deaths related to immunodeficiency had an AIDS-defining condition reported in the SIM. On the other hand, many cases that would have been attributed to HIV/AIDS based on ICD-10 codes registered in the basic cause were not related to HIV immunodeficiency by CoDE.

In recent years, CoDe has been implemented in clinical trials and cohorts, with the partial or complete use of its protocol, especially in European studies^12^, noting that the data collection instruments and the classification algorithm allow for an accurate evaluation of death, and that its coding system includes an adequate spectrum of causes of death in PLWHA^11^. In Brazil, two surveys used the CoDe methodology, both in the Southeast of the country^1^ ^,^ ^8^. The studies using CoDe reported a notable decrease in death rates due to immunodeficiency in PLWHA; however, they indicate that they are still the most frequent in Brazil^1^ ^,^ ^8^ and in some countries of Europe^9^ ^,^ ^11^, although “non-AIDS deaths” are increasing in this continent^1^ ^,^ ^14^ ^,^ ^15^ ^,^ ^20^. The reduction in HIV immunodeficiency deaths has been attributed to the effectiveness of current treatment regimens; while a large number of deaths due to immunodeficiency occurring shortly after starting ART has been associated with delays in diagnosis and treatment^1^ ^,^ ^15^ ^,^ ^19^.

In our cohort, despite most patients being on ART when entering the study, more than 80% of those who died were out of treatment (data not shown). This would explain why infections were the most frequent specific events among deaths, both related to immunodeficiency (76%) and unrelated (47%). We observed that tuberculosis was the most frequent infection among immunodeficiency-related deaths, in agreement with studies that point to it as the leading cause of death among opportunistic infections^7^, considering that HIV/tuberculosis coinfection accelerates the progression of both diseases^5^ ^,^ ^13^.

On the other hand, we highlight cancers (12%), external causes (15%), and cardiovascular diseases (11%) as important causes of death unrelated to immunodeficiency. These results are in line with the increasing trends of PLWHA deaths caused by violence, accidents^1^ ^,^ ^15^ ^,^ ^16^, and cardiovascular diseases^1^ ^,^ ^8^ ^,^ ^11^ ^,^ ^15^ ^,^ ^19^, both in developed countries^1^ ^,^ ^11^ ^,^ ^15^ ^,^ ^19^ as well as in Brazil^1^ ^,^ ^8^ ^,^ ^18^; and non-AIDS cancers^1^ ^,^ ^11^ ^,^ ^18^ ^,^ ^20^, now the main cause in some developed countries^19^. The rapid growth of external causes in PLWHA may be related to lifestyles, such as greater use of alcohol and illicit drugs^14^. In turn, the increase in survival after the introduction of ART has led to aging and prolonged exposure to low chronic immunity and inflammation, mechanisms implicated in carcinogenesis^1^ ^,^ ^14^ and in cardiovascular lesions in HIV patients^1^ ^,^ ^14^.

In epidemiological realities such as ours, with multiple causes of death in PLWHA^10^, reliable and standardized data on death are necessary so that studies can be compared and trends assessed over time. For the causa mortis to be correctly established, clinical information is essential, such as cardiovascular risk factors, drug use, coinfections, and viral load and CD4 values. When using ICD-10, death is attributed to HIV infection without considering clinical data of the case, and it is possible to overestimate the lethality of the disease^9^. In contrast, CoDe is based on the case study to establish the causal link with the diseases present at the time of death (see ReCoDe), and applies a classification based on the patient’s immune status (see RevCoDe)^10^.

However, we emphasize that we use CoDe partially because we do not review the cases. We only classify the deaths based on the causes in the death certificates, which are filled-out by several non-research professionals. This may have been a source of information bias. In contrast, the use of a database connection algorithm, RecLink III 2, allowed us to monitor patients’ vital status, preventing losses. Finally, we were able to classify all deaths as related or not to immunodeficiency and we found that the CoDe algorithm adds essential information for the classification of deaths in PLWHA. We found that less than a third of the cases would be related to AIDS-defining conditions based on the CDC list. On the other hand, 80% of the deaths would be attributed to HIV/AIDS by ICD-10, while 74% were attributed to HIV immunodeficiency by the CoDe algorithm.

Our findings emphasize the complexity of the epidemiological scenario in a developing country where opportunistic infections coexist with aging diseases, impressing multiple burdens on PLWHA health services. In this context, we observed that the CoDe system increased the chance of classifying deaths occurring in our population and to attribute them to immunodeficiency when compared to the list of AIDS-defining diseases, although it was more conservative than the ICD when relating the deaths to immunodeficiency. Our study exemplifies classification errors that can occur on a large scale. We recommend that the translated CoDe instruments be validated in field surveys to be used in studies on PLWHA mortality studies in Portuguese-speaking countries, as well as in epidemiological surveillance to subsidize programs and public policies.

## Footnotes

a Centre of Excellence for Health, Immunity and Infections, Department of Infectious Diseases. CoDe documents. Copenhagen: CHIP [cited 2017 Jul 15]. Available from: http://www.chip.dk/Tools-Standards/CoDe/Documents

b Additional materials available on: https://goo.gl/jf98w8
